# Sustained and Efficient Delivery of Antivascular Endothelial Growth Factor by the Adeno-associated Virus for the Treatment of Corneal Neovascularization: An Outlook for Its Clinical Translation

**DOI:** 10.1155/2024/5487973

**Published:** 2024-09-09

**Authors:** Mengzhen Xie, Lixiang Wang, Yingping Deng, Ke Ma, Hongbo Yin, Xiaolan Zhang, Xingye Xiang, Jing Tang

**Affiliations:** ^1^ Department of Ophthalmology West China Hospital Sichuan University, Chengdu 610041, China; ^2^ Beijing Institute of Ophthalmology Beijing Tongren Eye Center Beijing Tongren Hospital Capital Medical University Beijing Ophthalmology and Visual Sciences Key Laboratory, Beijing, China; ^3^ School of Life Science and Engineering Southwest Jiaotong University, Chengdu, Sichuan, China; ^4^ Georgia State University, Atlanta, GA 30302, USA

## Abstract

Corneal diseases represent 5.1% of all eye defects and are the fourth leading cause of blindness globally. Corneal neovascularization can arise from all conditions of chronic irritation or hypoxia, which disrupts the immune-privileged state of the healthy cornea, increases the risk of rejection after keratoplasty, and leads to opacity. In the past decades, significant progress has been made for neovascular diseases of the retina and choroid, with plenty of drugs getting commercialized. In addition, to overcome the barriers of the short duration and inadequate penetration of conventional formulations of antivascular endothelial growth factor (VEGF), multiple novel drug delivery systems, including adeno-associated virus (AAV)-mediated transfer have gone through the full process of bench-to-bedside translation. Like retina neovascular diseases, corneal neovascularization also suffers from chronicity and a high risk of recurrence, necessitating sustained and efficient delivery across the epithelial barrier to reach deep layers of the corneal stroma. Among the explored methods, adeno-associated virus-mediated delivery of anti-VEGF to treat corneal neovascularization is the most extensively researched and most promising strategy for clinical translation although currently although, it remains predominantly at the preclinical stage. This review comprehensively examines the necessity, benefits, and risks of applying AAV vectors for anti-VEGF drug delivery in corneal vascularization, including its current progress and challenges in clinical translation.

## 1. Introduction

**Figure 1:**
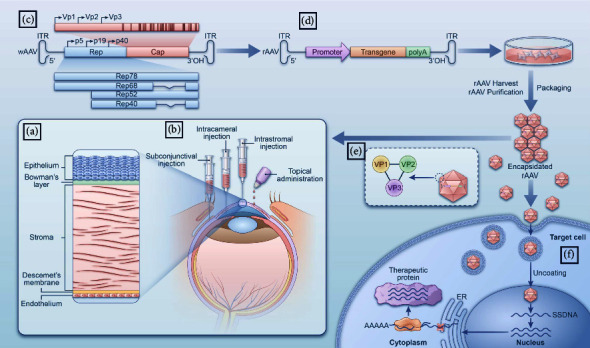
AAV transfer targeted gene to therapy for CoNV overview. (a) Five layers of cornea anatomy; (b) four different administration methods of AAV-mediated gene therapy for CoNV; (c) genome structure diagram of wAAV; (d) genome structure diagram of rAAV; (e) AAV capsid schematic; and (f) AAV vector transduction pathway.

The cornea, a unique tissue situated at the frontier of the globe, performs the critical task of light transmission. The normal cornea is avascular, but the limbus hosts a rich vascular network, primarily nourishing the peripheral cornea. For the central cornea, oxygen and nutrients are predominantly supplied through the aqueous humor, tears, air, and conjunctival vessels. Histologically, the cornea is divided from surface to back into the following five layers: the epithelium, Bowman's membrane, stroma, Descemet's membrane, and the endothelium (Figure 1(a)). Each layer is strategically arranged to reduce light scattering and maintain its transparency. The homeostasis of proangiogenic and antiangiogenic factors maintains the vascularity of the cornea [1]. In case of corneal neovascularization, this transparency is compromised as the neovascular tissues invade the central cornea from the limbus. Corneal neovascularization may occur either at the surface or in the deeper stromal layer, potentially leaving a residual ghost vessel wall. Directly exposed to the external environment, the cornea constantly encounters a variety of antigens and pathogens. Its ability to combat these invasive elements is crucial for clarity maintenance [2]. Corneal diseases are the second leading cause of blindness in developing countries and the fourth worldwide, affecting over 39 million people with complete blindness and 246 million people with visual impairment globally [3, 4].

Corneal neovascularization (CoNV), a prevalent cause of corneal blindness, results from chronic hypoxia and inflammation. Commonly associated with extended contact lens use, corneal infections, limbal stem cell deficiency, ocular surface inflammation, and eye injuries [5, 6], CoNV emerges due to an imbalance between proangiogenic and antiangiogenic factors. Pathologically, abnormal blood vessels from the limbus invade the corneal stroma, leading to scar tissue formation, edema, lipid deposition, and inflammation. CoNV is categorized into three types based on severity as follows: superficial neovascularization, vascular pannus, and interstitial or deep neovascularization. Beyond reducing visual acuity, CoNV compromises the cornea's immune privilege and heightens graft rejection risk in corneal transplant recipients [7, 8]. Vascular endothelial growth factor (VEGF) is a key angiogenesis mediator in the cornea, retina, and choroid [9, 10]. The past decade has witnessed a surge in commercialized anti-VEGF agents for neovascular retinal diseases, including proliferative diabetic retinopathy, wet age-related macular degeneration (wet AMD), and choroidal neovascularization, with new formulations explored to meet the challenge of multiple ocular drug barriers and achieve sustained release [11, 12]. For example, Reddy et al. used mesenchymal stem cell (MSC)-derived extracellular vesicles loaded with the anti-VEGF drug bevacizumab to reduce the frequency of intravitreal injection required for the treatment of diabetic retinopathy in a rat model. The extracellular vesicles-mediated drug delivery system, due to its similar composition to cells, maintains clarity in the vitreous body in the light path and can be considered for clinical applications in retinal diseases [13]. CoNV, a chronic and recurrent condition, necessitates long-term management. The corneal epithelium's tight junctions and rapid turnover due to lacrimation prevent the efficient delivery of the large molecular anti-VEGF drugs in its eye drops formulation to effectively reach the deep stromal layers of CoNV, which greatly limit the direct application of anti-VEGF drugs in its solution form [14]. Among the treatment methods mentioned above, gene therapy, particularly adeno-associated virus (AAV)-mediated gene delivery, shows immense potential, addressing the need for drug penetration and sustained release. In addition, AAV vectors offer unique advantages in treating CoNV due to their lower immunogenicity and high transduction efficiency. However, this novel strategy also faces challenges such as vector selection, delivery efficiency, and long-term safety.

By comparing the AAV-mediated anti-VEGF gene delivery method with existing treatment approaches, this paper aims to provide a more comprehensive perspective to assess the potential advantages and limitations of this new strategy in treating CoNV. This review explores the experiences and gaps in the clinical translation of AAV-mediated anti-VEGF transfer for CoNV treatment, underscoring the imperative for more comprehensive research to overcome this clinical challenge and propose possible future improvements.

## 2. Current Treatments of CoNV

### 2.1. Conventional Therapy for the Treatment of CoNV

Current pharmacological treatments for CoNV encompass topical steroids and nonsteroidal anti-inflammatory drugs. These medications primarily address the cornea's inflammatory conditions but offer limited benefit for established CoNV in inactive eyes. In addition, CoNV management includes argon laser photocoagulation, fine-needle diathermy (FND), photodynamic therapy (PDT), and amniotic membrane transplantation (AMT). However, these techniques exhibit restricted efficacy and are accompanied by potential side effects. Notably, none of these treatments directly target the molecular mediators of angiogenesis [15, 16].

### 2.2. Anti-VEGF Therapy for the Treatment of CoNV

Intravitreal injection of anti-VEGF drugs is the cornerstone of first-line therapy for retinal neovascular diseases. Commercially available anti-VEGF drugs include Lucentis® (Ranibizumab), Eylea® (Aflibercept), brolucizumab, conbercept (in China), and off-label bevacizumab (Avastin) [17–22]. To optimize effectiveness and duration, these drugs are either formulated for sustained release or administered at regular intervals, typically every 1-2 months [23]. The efficacy of anti-VEGF agents in treating retina and choroidal neovascularization has sparked extensive research into their potential for managing CoNV in both animal models and human patients.

The application of topical anti-VEGF agents for emerging, established CoNV, and graft rejection prevention in high-risk CoNV patients is debated. However, most studies endorse their positive role, influenced by variables such as formulation, frequency, dosage, and clinical indications [24]. Bevacizumab, for instance, whether applied in the formation of topical eye drops or by subconjunctival, limbal, and intrastromal injection has been shown to effectively inhibit or regress CoNV in animal models and patients [25–28]. Short-term application of bevacizumab, either topically or subconjunctivally, can mitigate CoNV severity without significant local or systemic adverse effects, offering an alternative treatment approach [25, 26, 29, 30]. Other anti-VEGF drugs like ranibizumab and aflibercept also demonstrate therapeutic benefits for CoNV patients [29, 30].

To counteract rapid excretion and turnover of topical applications, strategies such as frequent application or high-dose formulations are employed to increase drug exposure [31, 32]. Subconjunctival or intrastromal injections using microneedles extend drug effectiveness with minimal invasiveness and can be performed under topical anesthesia [27, 29, 33, 34]. For example, Britton et al. reported a case where refractory CoNV, against a backdrop of acne rosacea, completely regressed following two subconjunctival bevacizumab injections spaced two months apart, with no recurrence over a four-year follow-up [35]. Nevertheless, suboptimal dosing, exposure, and duration of drug may lead to compromised efficacy, and the relatively fast clearance of the large molecular anti-VEGF drugs through these conventional routes requires repeated administration or otherwise the therapy may be ineffective [27, 36–38]. Previous pharmacokinetic studies indicate bevacizumab applied as topical eye drops is unable to penetrate the intact cornea due to the epithelial barrier [39]. However, in vascularized cornea, the epithelial barrier is compromised, which enables the penetration of topical Bevacizumab into the stroma [40]. In addition, subconjunctival injection is found to be an efficient route for large molecular anti-VEGF agents to penetrate the intact corneal epithelium [40]. Despite the evidence supporting its penetration capacity, there are currently no data of the excretion profile and corneal concentrations of topically applied bevacizumab or other large molecules, indicating a knowledge gap in choosing an appropriate dose and frequency.

Although most studies suggest a significant reduction in CoNV after the application of anti-VEGF agents for several weeks, the effect is questioned as the ongoing irritation may extend for a much longer, and the recurrence of CoNV may occur when the drug is discontinued, such as in cases of herpetic keratitis or penetrating keratoplasty [41]. Although the long-term use of topical anti-VEGF agents by these administrative routes is generally well tolerated, several studies have reported complications associated with its extended use, including epithelial lesions, stromal matrix thinning, and corneal perforation. The repeated administration of topical anti-VEGF is both bothersome and expensive, which may be the major obstacle for its clinical application. Currently, no formulations of anti-VEGF agents have been approved for the treatment of CoNV, and innovative drug delivery routes such as gene therapy by AAV are promising alternative to achieve sustained drug release.

## 3. Structure, Function, and Immunogenicity of AAV-Mediated Transfer and Vector Selection

Gene therapy refers to the use of viral and nonviral vectors for transferring nucleic acids into cells to correct cell dysfunction or restore normal cell function, which has been actively explored in many medical fields [42]. The cornea, as the frontier window of the eye, possesses unique conditions for gene therapy, including its relatively immune-privileged state, superficial location for easy access, and its transparent nature that allows for real-time tracking of marker molecules in animal studies [43, 44]. Moreover, the corneal stroma contains nondividing corneal fibroblasts and keratocytes, ensuring that the AAV episomal DNA will not be diluted by mitosis. Gene-based antiangiogenic therapy is an emerging strategy that has been explored in both retina neovascular diseases and CoNV [16, 45]. At present, the recombinant viral vectors used in gene therapy mainly include adenovirus, retrovirus, lentivirus, adeno-associated virus (AAV), and herpes simplex virus [46]. Recombinant adeno-associated virus (rAAV) has become the preferred live viral vector for delivering genetic materials to target human diseases, which involves processes of gene augmentation, gene deletion, and/or gene editing. The AAV genome is a linear single-stranded DNA (ssDNA) with a size of approximately 4.7 kb. It is composed of an internal region that includes three promoters (P5, P19, and P40), two open-reading frames (ORFs), and rep gene encoding replication proteins (Rep78, Rep68, Rep52, and Rep40) on the left side (Figure 1(c)), and structural proteins (VP1, VP2, and VP3) encoded by the cap gene on the right side (Figures 1(c) and 1(e)). These regions are flanked by two inverted terminal repeats (ITRs) [47]. As a tool for gene therapy, AAV is nonpathogenic, low immunogenic, able to transfect multiple types of cells, and expressed in transfected host cells for a long time, unaffected by cell differentiation.

Worthy of mentioning is that AAV belongs to the parvovirus family, which requires coinfection with other viruses, mainly adenoviruses, to replicate [48]. AAV can infect both dividing and nondividing cells, and it seldom integrates into the host cell's genome. Rather, it persists within the cells as episomal DNA. This attribute is particularly advantageous for systemic gene delivery [48].

Currently, different AAV serotypes have not been found to be associated with any known diseases in humans, indicating good safety [49]. AAV produces relatively mild innate and adaptive immune responses, in part by allowing the sustained expression of therapeutic genes while preventing loss of its viability [50]. Currently, AAV has been used in clinical trials to treat a variety of diseases, including hemophilia [51], central nervous system [52], eye [53], muscle [54], heart [55], diseases of digestive system [56], and acquired immune deficiency syndrome (AIDS) [57]. These trials have repeatedly highlighted the safety, efficacy, and versatility of AAV as a clinical tool, and its potential as a platform for a broad range of future therapies [58]. To date, two AAV-based gene therapies, luxturna and zolgensma, have been approved for the treatment of RPE65-related Leber congenital amaurosis [59] and spinal muscular atrophy [60].

Currently, AAV-based gene therapy shows promising results for clinical translation in the field of ophthalmology, and AAV-based therapies have separately completed Phase II clinical trials for diabetic macular edema (NCT04418427) and Phase I clinical trials for wet age-related macular degeneration [61]. AAV vectors have been used to transfect corneal epithelial cells with antiangiogenic genes such as endostatin and angiostatin, which successfully reduce CoNV effects in animal models [16, 62]. Recombinant adeno-associated viruses (rAAVs) have shown great promise due to their low immunogenicity and genotoxicity profiles, broad tropism, high in vivo transduction capacities, and long-term efficacies [63, 64]. The AAV vector is an effective and safe means to deliver genes to the cornea [44]. AAV-based gene delivery has been able to transfect the corneal stroma and endothelium without apparent toxicity both in vivo and in vitro [16, 44, 65–67]. Molecular cloning of the AAV gene, originally distinguished from serology, has identified hundreds of unique AAV strains in many species [68]. Although different AAV serotypes exhibit broad host tissue tropism, almost every organ or tissue has preferred serotypes to target [65]. In bovine corneal experiments, results have shown that rAAV2 vectors controlled by functionalized contact lenses can efficiently transduce corneal cells without causing significant immune responses. AAV2 effectively transduces stromal keratocytes in the cornea [69]. In rabbit eye experiments, rAAV2 can deliver genes directly to the ocular surface epithelium via subconjunctival injection, maintaining sustained and high levels of gene expression in vivo to inhibit neovascularization [70]. In addition, in rabbits, AAV5-mediated gene delivery through either intracorneal stromal injection or topical application on the exposed corneal stroma successfully transduced corneal cells, showing effective transduction without cytotoxicity [67, 71, 72]. In fresh ex vivo porcine eyes, studies revealed that AAV8-GFP induced a very mild immune response, with only a slight capsid antibody response detected in a single animal. Upon injecting AAV8 into the corneal stroma, AAV8 efficiently transduced various corneal cells, including stromal keratocytes, without causing significant off-target effects or adverse reactions [73]. For fresh human limbal and central corneal epithelial cells, among AAV2, AAV4, and AAV6, AAV6 exhibited the highest transduction efficiency, followed by AAV4 and then AAV2. Furthermore, AAV6 led to transduction of corneal stroma and endothelial cells two days poststromal injection [74]. In mice, results from subconjunctival injections of AAV2, AAV6, and AAV8 indicated that AAV6 was primarily localized to endothelial cells with occasional low-level expression in the stroma [75]. In contrast, AAV8 resulted in strong stromal transduction without significant expression in the epithelium or endothelium. The transduction efficiency of AAV8 and AAV6 in the cornea was significantly higher than that of AAV2 [75]. Sharma et al. evaluated the tropism and relative transduction efficiency of AAV6, AAV8, and AAV9 serotypes when topically applied to in vivo mouse corneas and ex vivo human corneas. All three serotypes successfully transduced both mouse and human corneas, with transduction efficiencies in the order of AAV9 > AAV8 > AAV6. AAV9's transduction efficiency was 1.1–1.4 times higher than AAV8 (*p* > 0.05) and 3.5–5.5 times higher than AAV6 (*p* < 0.01). Corneas exposed to any of the three serotypes showed no significant cytotoxicity or inflammatory responses, indicating that these AAV serotypes are safe for corneal gene therapy [66]. To sum up, because there are few studies on AAV9 in cornea, AAV2 and AAV8 have been found as the two most efficient serotypes for transducing corneal stromal cells, compared with other commonly tested vectors such as AAV6 and AAV9 [75–78]. The clinical use of AAV2 vectors may be compromised by the presence of AAV2 neutralizing antibodies in humans, and this concern can be minimized by using nonhuman primate-derived AAV8 serotypes [79]. It has also been shown that AAV8 transduces most efficiently in the mouse corneal stroma [80], whether administered locally after epithelial removal [66], or injected into the corneal stroma [76]. In studies of chimeric AAVs, testing AAV2/1, AAV2/2, AAV2/5, and AAV2/8 serotypes showed that AAV2/8 was most effective for transducing corneal stromal cells following intracorneal injection, followed by AAV2/1. AAV2/2 and AAV2/5 exhibited lower transduction efficiencies for gene transfer in both mice and humans [76]. Intracameral injection of AAV2/9 in mice effectively transduced corneal endothelial cells [81]. Another study indicated that primary cultures of human corneal fibroblasts tested with AAV2/6, AAV2/8, and AAV2/9 vectors showed that all three serotypes transduced human corneal fibroblasts. The transduction efficiencies were in the order of AAV2/6 > AAV2/9 > AAV2/8. Furthermore, these serotypes did not induce significant cell death, suggesting they are safe for corneal gene therapy [65]. In addition, new recombinant vectors, such as the AAV8/9 chimeric (8G9) capsid reported by Vance et al. result in more than a 10-fold supraphysiological increase of gene transfection to human corneal stroma (ex vivo) and is another strategy to improve its transduction efficiency [82]. Similar results were observed with AAV8G9-IDUA gene injection into the canine corneal stroma [83].

## 4. Pathophysiology and Delivery Methods of AAV-Mediated Anti-VEGF Therapy

Recombinant AAV (rAAV) is constructed by replacing the rep gene and cap gene of AAV with corresponding target genes, and the expression cassette includes a promoter, the target gene, and a polyA tail (Figure 1(d)). The rAAV package is recognized and endocytosed by the target cells via specific surface receptors. Once inside the nucleus, the rAAV uncoats and the rAAV genome is released, which is then replicated into double-strand DNA to enable transcription [84] (Figure 1(f)).

AAV-mediated anti-VEGF therapy for CoNV can be delivered via four potential routes, including (1) topical application [85, 86], (2) intrastromal injection [80, 83, 87], (3) intracameral injection [81, 88–90], and (4) subconjunctival injection [62, 75, 80, 91, 92] (Figure 1(b)), which all have been tested with preliminary data available. No deleterious consequences associated with AAV vectors were observed so far with these administration routes [66, 78, 85] (Table 1). For the treatment of CoNV, topical application seems to be the most attractive due to its easiest way to delivery drugs and the noninvasive nature. However, the penetration capacity of its target protein as massive immunoglobulin across the corneal epithelium has been questioned. In the condition of CoNV the integrity of corneal epithelial tight junctions is compromised, therefore allowing the penetration of bevacizumab across the corneal epithelial barrier to a certain extent [93], AAV gene delivery via this pathway is still minimal without prior corneal epithelial debridement [66, 94, 95]. Using polymers or other strategies to improve the permeability of AAV vectors in vivo may be a solution [96–98]. The damage caused by epithelial curetting after alcohol burns may mimic various corneal injuries, and rAAV can be directly exposed to corneal stromal cells by eye drops compared to eyes with an intact epithelial barrier [87]. However, topical administration of AAV vectors has the risk of systemic exposure when the eye drops enter the nasal cavity through the nasolacrimal duct. Despite limited data on the risk of induction of systemic immune reactions and development of neutralizing antibodies, there is reasonable concern over the issue and more studies are needed to confirm its safety. The injection of AAV vectors into the corneal stroma and anterior chamber are efficient ways to target different corneal layers [76, 78, 88–90]. Intrastromal injection is the most popular way to transfect the corneal stroma for the treatment of CoNV and intracameral injection is a feasible way to transfect the corneal endothelial cells [76, 90]. Intrastromal injections appear to be well tolerated and nontoxic, which can be conducted in the outpatient setting under topical anesthesia, with the invention of minimally invasive microneedles. In general, intrastromal injection of AAV vectors results in more efficient gene transfer compared with topical application and subconjunctival injection [80]. The delivered AAV vectors almost remain in the ocular compartments, with minimal release into the tear film under the test conditions, demonstrating low risk of systemic exposure [99, 100]. The benefits of intracameral injection closely parallel those observed with stromal injections. Notably, intracameral injection facilitates the transfection of the corneal endothelium and trabecular meshwork tissues. Nevertheless, this procedure is associated with potential risks, including endothelial damage and an increase in intraocular pressure. Compared to the targeted delivery of AAV vectors via intrastromal/intracameral injection, subconjunctival injection results in a broader distribution and may even target the posterior globe with few side effects [101]. AAV vectors injected into the subconjunctival space are distributed over a large periocular space and have been used to deliver drugs to different target tissues, including the retina and optic nerve [102–104]. Subconjunctival injection of the AAV vectors results in gene transfer to the subepithelial muscles and soft tissues of the eye, suggesting that the vectors can easily infiltrate the systemic circulation in this way, which are then captured by the liver [92]. As with intrastromal injection, AAV injected into the subconjunctival space are rarely shed into tears [75]. Despite these features, delivery of AAV vectors via subconjunctival injection remains poorly characterized and no analysis comparing the efficacy and specificity of different serotypes has been performed. In particular, the pharmacokinetic analysis of corneal exposure of anti-VEGF drugs via subconjunctival injection is poorly understood, thus limiting its application in the treatment of CoNV.

## 5. Safety and Immune Response of AAV-Mediated Anti-VEGF Therapy

The eye is a highly compartmentalized organ and its local environment is anatomically and physiologically isolated by the presence of the blood-retinal barrier and the blood-aqueous barrier [105, 106]. In addition, a phenomenon called anterior chamber associated immune deviation has been described in the eye, which tolerates immunogenic antigens by a variety of mechanisms to maintain its state of immune privilege [107]. Thus, in the context of gene transfer, it has long been assumed that the eye has a low risk of activating innate and adaptive immune responses after viral vector injection [108].

Previous studies injecting AAV vectors into the vitreous cavity or the subretinal space support the good tolerability of the eye for these foreign materials [109–111]. The cornea is also endowed with immune exemption in the physical condition, and a complex set of unique structural and functional features enable it to modulate immune responses and prevent immune damage [2]. The normal human cornea, except the limbus, is devoid of blood and lymphatic vessels, which limits antigen presentation and immune cell infiltration [112]. However, CoNV is a special pathological condition regulated by a variety of mediators, and one of the key regulators is VEGF, a growth factor produced by corneal epithelial cells, macrophages, and fibroblasts [8, 113, 114]. In response to VEGF, blood and lymphatic vessels infiltrate the cornea and disrupt the normal immune-privileged state of the cornea [2, 115]. Host immune responses are important considerations of gene therapy because inflammatory responses may reduce treatment effectiveness and cause irreversible damage to tissues. Although AAV is characterized by low immune responses and low levels of toxicity, AAV-mediated toxicity may vary in different tissue types [66]. In the context of AAV-mediated gene therapy to the ocular tissues, several studies have shown that there is much less immune response in the eye compared with systemic administration [116, 117]. For example, injection of low-dose AAV virus vector into mice cornea induced no early innate immune response but only injection-related tissue damage [80]. The immunogenicity of AAV vectors is dose dependent, and low vector doses are more likely to induce mild inflammation that can be controlled and does not result in complete loss of transgene expression [100, 118, 119]. Only the animals that received the highest dose developed capsid-neutralizing antibodies after the injection, compromising the efficiency of treatment [83]. However, depending on the total dose of the delivery vehicle, the inflammatory response can be detected regardless of the route of administration [120].

Up to 90% of people have asymptomatic infection with AAV during their lifetime [121]. As a result of the subclinical infection with wild-type adeno-associated virus (wAAV) in infancy, most individuals develop anti-AAV neutralizing antibodies, and these preexisting neutralizing antibodies may limit the efficiency of gene transfer depending on the target cell type, route of administration, and choice of serotype [122]. Fortunately, the immune-privileged state of the eye largely reduces such immune reactions and enables more efficient delivery of genetic materials. The success of clinical ocular gene transfer has been driven by the characteristic immunological privilege, the fact that circulating antibodies against AAV capsids are usually not present in the eye, and the relatively low vector doses required to achieve the therapeutic effect [123–125]. Another potential problem associated with AAV-mediated transfer of anti-VEGF therapy to treat CoNV is the potential immune reaction against the target recombinant protein expressed. The whole antibody containing the fragmented crystalline (Fc) domain, such as full-length bevacizumab, may induce unwanted immune responses in the eye [126]. In turn, a single-chain fragment variable (ScFv) antibody, which lacks the Fc domain, has been proposed as a safer alternative. ScFv and immunoglobulin G1 (IgG1) have obvious therapeutic effects on mouse CNV. Importantly, anti-VEGF ScFv expressed from the AAV vector also showed a significant benefit of inducing low immune reaction, providing valuable preclinical data for future translation into clinics [127].

## 6. Explorations of AAV-Mediated Anti-VEGF Therapy for CoNV in Animal Experiment

Frequent intravitreal injection of anti-VEGF drugs increases the burden on patients and the risk of associated adverse events. AAV-mediated delivery of anti-VEGF agents theoretically provides a long-term source of antiangiogenic proteins after a single dosing [127]. Genomic disruption of VEGF-A using AAV delivery of clustered regularly interspaced short palindromic repeats (CRISPR)-Cas9 has the potential to permanently suppress aberrant angiogenesis [128]. So far, AAV has been shown to be safe in some clinical trials, with a much lower risk of insertional mutations compared to lentiviruses [129]. AAV vectors have also been tested in several landmark clinical trials of gene therapy in the eye, including DME and wet AMD [50, 130]. Despite the successful application of AAV-mediated gene therapy in the posterior segment of the eye, it has hardly ever completed phase I clinical trials for any anterior segment diseases.

In mice CoNV, intrastromal injection of a single dose of recombinant AAV to mediate KH902 (Conbercept) expression resulted in persistent expression of the target protein in the cornea and regression of CoNV after 3 months without remarkable adverse effects [131]. In comparison, Conbercept only had a lasting effect for 10–14 days after a single intrastromal administration [80]. In a cautery-induced neovascularization model in rabbit eyes, rAAV transduces the soluble VEGF receptor 1 (sFlt-1) gene and can reduce the development of corneal neovascularization by 36% [132]. AAV is also used to carry a number of other molecules, such as HLA-G codon, miR-204, Decorin, Endostatin, and angiostatin, which show good potential for inhibiting corneal neovascularization [62, 67, 70, 85, 99, 133, 134] (Table 2). While the literature on using AAV for transferring VEGF-related genes to treat CoNV is limited, it is promising to observe that in animal studies where AAV has been employed as a vector for transferring various target genes to cornea, no significant side effects related to AAV transfection have been reported.

## 7. Safety/Adverse Events/Delivery Method Challenges

Using AAV-mediated gene therapy for corneal disease is a promising strategy, but as with any medical intervention, it has potential side effects. While specific information on adverse effects associated with the treatment of corneal disease is limited, we can gain some general insights from the widespread use of AAV in gene therapy. AAV-mediated gene therapy is relatively safe, especially in diseases that require local or systemic delivery at lower vector doses. However, the long-term safety of AAV-based gene transfer is still being studied, as more data from ongoing clinical trials are needed to fully understand.

Hepatotoxicity including raised levels of liver enzymes such as ALT (alanine aminotransferase) and AST (aspartate aminotransferase) is the most frequently observed adverse event associated with AAV therapy. This has been documented in some preclinical animal studies as well as in clinical trials. Current evidence indicates that high doses of AAV therapy, especially in the presence of pre-existing liver disease, can lead to severe liver toxicity [135]. In a cynomolgus macaque utero model transfer long-term factor IX using AAV, a transient neutral immune response was observed but no adverse reactions and clinical toxicity [136].

In preclinical studies on animal models, there have been reports of integration of the AAV vector transgene into the host genome leading to clonal expansion and other adverse events [137, 138]. However, these observations have not been reported in human trials so far. Side effects observed in humans following AAV therapy encompass a range of conditions including thrombotic microangiopathy, hepatocellular carcinoma, acoustic neuroma, frontal/intracranial hemorrhage, acute ischemic stroke, cell infiltration, thrombocytopenia, proprioceptive deficits, and ataxia, as well as inflammation [135]. However, the precise mechanisms underlying these reactions remain incompletely understood. Furthermore, the long-term risks associated with these treatments are still under investigation.

As mentioned above, when AAV gene therapy is applied to the cornea, which usually require lower doses of the viral vector compared to whole-body treatments, the viral vectors are typically injected directly into a specific area, such as the intrastromal injection. This small dose and targeted approach limit the spread of the vector to other parts of the body, reducing the risk of systemic side effects. In contrast, whole-body treatments often require systemic delivery, where the vectors can distribute throughout the body, potentially affecting multiple tissues and organs. The eye is considered an immune-privileged site, meaning it is somewhat isolated from the body's immune system. This unique aspect reduces the likelihood of immune reactions, which is a common cause of side effects, against the viral vectors or the therapeutic genes they carry. However, it is important to note that while the incidence of side effects may be lower, they are not completely absent in ocular gene therapy. Potential side effects can include inflammation, immune responses, and damage to ocular structures, but these are generally less severe and easier to manage than systemic side effects.

It is worth mentioning that limited delivery capacity is a major disadvantage of viral vectors. However, in practical applications, some researchers have proposed some practical and effective solutions, for example, a Dual-AAV has been developed to expand the carrying capacity of the virus vector, which has a wide application prospect [139, 140].

## 8. Conclusions and Future Perspectives

In the past decade, studies of the application of topical anti-VEGF agents have provided supportive data of their efficacy to inhibit and regress CoNV. However, the presence of the corneal epithelial barrier greatly restricts the penetration of the large molecular immunoglobulin drugs into the corneal stroma and compromises their therapeutic effects. The reliance on frequent application of high dose anti-VEGF eye drops is both expensive and bothering, which may be the major obstacle for its clinical translation. AAV-mediated transfer is a promising strategy for the sustained delivery of anti-VEGF agents to treat CoNV. Although theoretically a single dose of administration may provide sustained protection, there is no long-term observational study that supports its efficacy over years. The viral vectors are most commonly administrated by intrastromal injection, which presents with minimal tissue damage and well-targeted exposure to the corneal stroma. However, topical administration and subconjunctival injection are alternative routes but the risk of systemic exposure and nonspecific transfection limits their application in CoNV. Preliminary data indicate that delivery to AAV-mediated anti-VEGF to the corneal is well tolerated and minimal immune reactions are evoked due to the immune-privileged state of the cornea, which seems to be an advantage over other tissues. However, currently no in-human data of any recombinant vectors for corneal diseases are available and its pace of clinical translation seems to fall behind posterior segment diseases, despite the easier way to access cornea than the retina.

The major gaps in the translation include (1) the unknown corneal pharmacokinetic profile of AAV-mediated anti-VEGF drugs delivered via intrastromal or topical administrations; (2) the fast turnover of the corneal epithelium, which is a major route of VEGF responsible for CoNV; (3) the question of the efficacy of anti-VEGF therapy for regressing established CoNV; (4) the challenge of inhibiting CoNV in the presence of limbal stem cell deficiency, which is a common condition associated with CoNV, (5) AAV-mediated delivery of anti-VEGF agents targets only one pathway that promotes angiogenesis, whereas CoNV is regulated by multiple pathways [16], and it may be possible to use AAV to simultaneously package multiple anti-angiogenesis genes in the future, and (6) in preclinical gene therapy, establishing robust dose-response relationships tailored to the specific disease being treated, along with evaluating potential toxicity using the most suitable viral vector serotype, can enhance the safety and efficacy of human AAV gene therapy. In conclusion, AAV-mediated delivery of anti-VEGF agents is a promising therapy for CoNV, but researchers need to fill the gap of unknown to boost its clinical translation.

## Figures and Tables

**Table 1:** Comparison of delivery modes of AAV gene therapy for CoNV.

	Topic application	Subconjunctival injection	Intrastromal injection	Intracameral injection
Advantages	Models for corneal epithelial injury are relatively advantageous; noninvasive and low risk	Simple to operate; low risk; rarely shed in tears	Well tolerated; not released into the tear film; relatively concentrated distribution	Well tolerated; not released into the tear film; relatively concentrated distribution
Disadvantages	Lower penetration efficiency; may be diluted quickly by tears	Relatively distribution widespread	More invasive, potential for tissue damage	Higher risk of intraocular pressure increase; potential for endothelial damage
Eye distribution	Confined to the epithelium and stromal boundaries	Distributed to the posterior segment of the eyeball and the periocular muscles	Distributed to the anterior and posterior segments of the eyeball and the subconjunctival area	Expressed in corneal endothelium, trabecular meshwork but not in stroma
Tissue distribution	—	Penetrate into the systemic circulation and absorbed mainly by the liver	—	—

**Table 2:** AAV gene therapy for treatment of corneal neovascularization.

Target gene	Virus serotype	Delivery	Subject	Model	Dose	Follow-up duration	Result	Serious side effect
Decorin [85]	rAAV5	Topical(with epithelium removed)	Rabbit	Implant VEGF pellet into cornea stroma pocket	A single topical AAV5 titer (100 *μ*l; 5 × 10^12^ vg/ml) (one day after VEGF pellet implantation)	2 weeks	Significant reduction in corneal neovascularization by 63 ± 6.3% on day 14, with no major side effects	None
Decorin [67]	rAAV5	Topical apply	Rabbit	—	50 *μ*l titer; (6.5 × 10^12^ vg/ml)	6 months	AAV5–Dcn gene therapy caused no significant toxicity to the cornea	None
HLA-G codon [99]	rAAV8G9	Intrastromal injection	Rabbit	Alkali burn	Injection of 5 × 10^10^ viral genomes (seven days after corneal wounding)	8 weeks	Inhibited *α*-SMA expression and near complete inhibition of corneal vascularization	None
Endostatin [70]	rAAV2-CMV	Subconjunctival injection	Mice	Chemical cauterization	5 *μ*l; 2.5 × 10^7^ viral particles (two weeks before chemical cauterization)	8 months	Significantly inhibit of angiogenesis	None
Angiostatin [62]	rAAV	Subconjunctival injection	Rat	Alkali burn	5 *μ*l; 1 × 10^10^ viral particles (three weeks before alkali injury)	16 weeks	Reduced alkali burn-induced corneal angiogenesis	None
KH902 [131]	rAAV2/rAAV8	Intrastromal or subconjunctival injection	Mice	Alkali burn or suture procedures	4 *μ*l; 1.6 × 10^10^ genome copies (GCs)/8 × 10^8^ GCs	12 weeks	Significantly inhibit of corneal neovascularization	None
miR-204 [133] (a microRNA)	rAAVrh.10	Intrastromal or subconjunctival injection	Mice	Alkali burn	4 *μ*l; 3.6 × 10^10^ GCs	2 weeks	Partial inhibit of corneal neovascularization	None
sFlt-1 [132]	rAAV2-CMV	Intra-cameral injection	Rat	Cautery-induced	2 *μ*l; 4 × 10^11^particles/ml	16 weeks	Reduced the development of corneal neovascularization by 36%	None

HLA-G: human leukocyte antigen G. Plgf1-de: a placental growth factor 1 variant, KH902Conbercept, also known as KH902, is an anti-VEGF drug, which is a soluble recombinant protein.

## Data Availability

The data that support the findings of this study are included within the article.
